# Oral 25-Hydroxycholesterol Suppresses 4T1 Tumour Growth in an Immune-Context-Associated Manner

**DOI:** 10.3390/ijms27219735

**Published:** 2026-10-30

**Authors:** Shuo Liang, Jingyao Xu, Zining Wang, Yuhao Liu, Yanlei Xu, Chang Yao, Zhiyuan Zhu

**Affiliations:** 1 Affiliated Hospital of Nanjing University of Chinese Medicine, Nanjing 210029, China; 18260026992@163.com (S.L.); xuehao29@126.com (Y.X.); 2 School of Chinese Medicine, Nanjing University of Chinese Medicine, Nanjing 210023, China; 3 The First Clinical Medical College, Nanjing University of Chinese Medicine, Nanjing 210029, China

**Keywords:** 25-hydroxycholesterol, triple-negative breast cancer, 4T1, CD8-positive T cells, tumour microenvironment, macrophage polarisation, AMPK/STAT6 signalling, immune-context association, nude mice

## Abstract

Triple-negative breast cancer (TNBC) is an aggressive subtype with limited targeted-treatment options. 25-Hydroxycholesterol (25-HC) is an oxysterol involved in metabolic and immune regulation, but its antitumour effects remain context dependent. We examined whether oral 25-HC suppresses 4T1 tumour growth in immunocompetent BALB/c mice and whether host immune status modifies the response. Mice bearing 4T1 tumours received oral vehicle or 25-HC (1 or 10 mg/kg) daily for 14 days (*n* = 6 per group). 25-HC reduced endpoint tumour volume from 1510.6 ± 235.4 mm^3^ (vehicle) to 1147.2 ± 243.1 and 866.7 ± 206.6 mm^3^ (adjusted *p* = 0.027 and 0.0004); the 10 mg/kg dose also reduced tumour weight and Ki-67 positivity, increased CD8-positive T-cell infiltration and the granzyme B (GZMB)-positive fraction, reduced M2-like macrophages and raised the M1/M2 ratio. Tumour-tissue adenosine monophosphate-activated protein kinase (AMPK) signalling was altered, with p-AMPK/AMPK increased to 1.23- and 1.57-fold of control and p-STAT6/STAT6 decreased to 0.81- and 0.46-fold. Serum 25-HC increased with dose, while intratumoral 25-HC was lowest at 10 mg/kg; no dose-associated reduction was observed in BALB/c nude mice. Oral 25-HC thus suppressed 4T1 tumour growth with immune-context-associated features; however, the treatment-by-host interaction did not reach statistical significance (*p* = 0.06), and immune-context association of the response remains suggestive rather than established. The reciprocal serum–tumour pattern, a descriptive observation from a single time point whose cause remains unclear, generates a testable hypothesis of immune-cell-associated 25-HC handling.

## 1. Introduction

Triple-negative breast cancer (TNBC) is a clinically important subgroup of breast cancer defined by the absence of oestrogen receptor (ER), progesterone receptor (PR) and human epidermal growth factor receptor 2 (HER2) expression. Compared with receptor-positive disease, TNBC is more frequently associated with rapid progression, early relapse and visceral metastasis, and chemotherapy remains the backbone of systemic treatment [1]. Although immune-checkpoint blockade and antibody–drug conjugates have expanded the therapeutic landscape, resistance and tumour heterogeneity remain major barriers [1]. Metabolic vulnerabilities that can be coupled to immune modulation may therefore represent useful therapeutic opportunities.

Cholesterol and its derivatives play broad roles in membrane organisation, cell signalling and immune regulation [2]. Oxysterols, including 25-hydroxycholesterol (25-HC), are endogenous cholesterol metabolites that regulate sterol sensing and immune signalling through cell-type-specific mechanisms [3]. Their effects are strongly context-dependent: the same metabolite can influence tumour cells, macrophages and lymphocytes differently according to local concentration, receptor expression and metabolic demand. In tumour-associated macrophages (TAMs), for example, lysosomal accumulation of 25-HC activates AMPKα and signal transducer and activator of transcription 6 (STAT6), driving an immunosuppressive transcriptional programme that impairs T-cell-mediated tumour control [4]. At the systemic level, breast cancer-derived extracellular vesicles carrying miR-9-5p induce hepatic cholesterol 25-hydroxylase (CH25H) expression and elevate circulating 25-HC, driving Kupffer-cell M2 polarisation and creating an immunosuppressive niche that promotes liver metastasis [5]. Other oxysterols, such as 27-hydroxycholesterol, have likewise been linked to breast cancer metastasis [6]. Conversely, 25-HC also serves as a metabolic fuel for T cells: tumour-derived factors induce ATF3 in cytotoxic T lymphocytes (CTLs), suppressing CH25H and depleting intracellular 25-HC, which enhances effector trogocytosis and drives CTL exhaustion and apoptosis; restoring CH25H expression in CAR-T cells suppresses trogocytosis and improves CTL survival and antitumour efficacy [7]. Lymphatic-derived oxysterols have also been shown to promote antitumour immunity and responses to immunotherapy [8]. Together, these findings indicate that the local concentration of 25-HC is set by a dynamic balance between production (mainly by macrophages [9]) and consumption (by activated T cells), and that this balance may be the mechanistic basis of the context-dependent behaviour of 25-HC [3,4,7].

The 4T1 model is an immunocompetent, syngeneic mouse model of TNBC that closely reproduces key features of human breast cancer and permits simultaneous evaluation of tumour growth and host immune responses [10]. In the present study, we asked whether exogenous 25-HC suppresses 4T1 tumour growth in immunocompetent BALB/c mice and whether the response is accompanied by changes in tumour-cell proliferation, cytotoxic CD8-positive T cells, macrophage polarisation and cholesterol-related signalling that would be consistent with an immune-mediated mechanism.

This study tested the hypothesis that 25-HC suppresses TNBC growth via coordinated effects on tumour metabolism and the immune microenvironment, and examined whether the antitumour response differed between immunocompetent BALB/c mice and T-cell-deficient BALB/c nude mice. Because nude mice have broader immunological abnormalities in addition to T-cell deficiency, this comparison was designed to probe immune-context association rather than to establish CD8-positive T-cell necessity.

## 2. Results

### 2.1. 25-HC Reduced Tumour Burden in Immunocompetent 4T1-Bearing Mice

**Figure 1:**
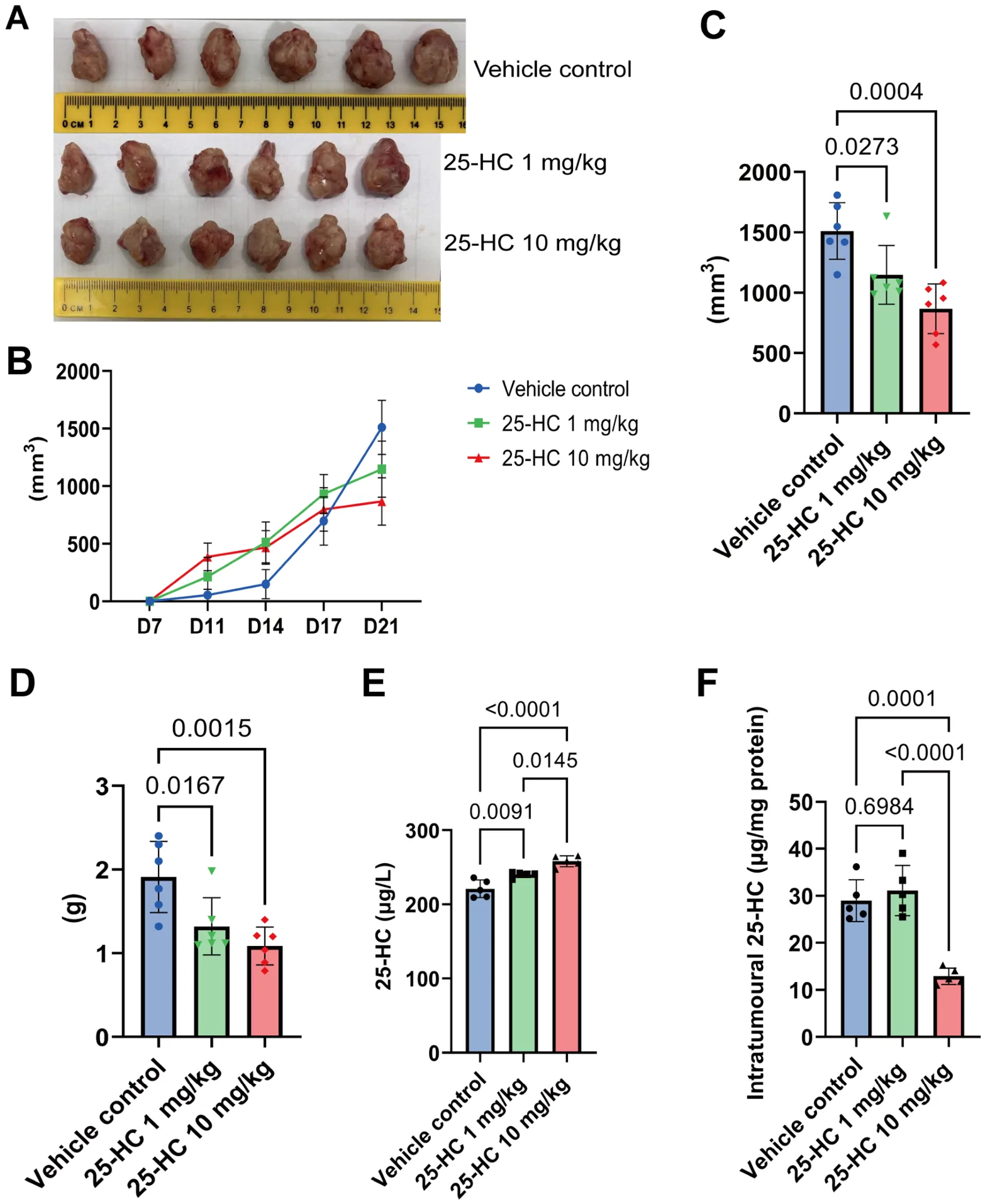
25-HC reduced 4T1 tumour burden and produced a reciprocal serum–tumour 25-HC distribution. (**A**) Tumour images at endpoint; (**B**) tumour growth curves (days 7–21); (**C**) endpoint tumour volume (n = 6 per group); (**D**) tumour weight at endpoint (n = 6 per group); (**E**) serum 25-HC by competitive ELISA (n = 5 per group); (**F**) intratumoral 25-HC normalised to total protein (n = 5 per group). Bars show mean ± SD. One-way ANOVA with Dunnett’s test vs. vehicle: (**C**) adjusted *p* = 0.027 and 0.0004; (**D**) adjusted *p* = 0.0167 and 0.0015 for 1 and 10 mg/kg, respectively. (**E**,**F**) One-way ANOVA followed by Tukey’s HSD test: (**E**) adjusted *p* = 0.0091 (1 mg/kg), *p* < 0.0001 (10 mg/kg) and *p* = 0.0145 (1 vs. 10 mg/kg); (**F**) adjusted *p* = 0.6984 (1 mg/kg), *p* = 0.0001 (10 mg/kg) and *p* < 0.0001 (10 vs. 1 mg/kg).

Tumour growth curves and representative endpoint tumour images are shown in Figure 1A,B. At the final recorded time point, mean tumour volumes were 1510.6 ± 235.4 mm^3^ (vehicle), 1147.2 ± 243.1 mm^3^ (1 mg/kg 25-HC) and 866.7 ± 206.6 mm^3^ (10 mg/kg 25-HC), corresponding to endpoint-derived reductions of 24.1% and 42.6% relative to the vehicle mean (one-way ANOVA: *F*(2,15) = 11.93, *p* = 0.0008; Dunnett’s adjusted *p* = 0.027 and *p* = 0.0004 for 1 and 10 mg/kg vs. vehicle, respectively; Figure 1C). Baseline body weight did not differ among groups at randomisation (one-way ANOVA: *F*(2,15) = 2.03, *p* = 0.165).

Tumour weight showed the same direction of effect: 1.91 ± 0.43 g, 1.32 ± 0.34 g and 1.09 ± 0.23 g in the vehicle, 1 mg/kg and 10 mg/kg groups, respectively (one-way ANOVA: *F*(2,15) = 9.30, *p* = 0.0024; Dunnett’s adjusted *p* = 0.0167 and *p* = 0.0015 for 1 and 10 mg/kg vs. vehicle, respectively; Figure 1D).

### 2.2. Oral 25-HC Raised Serum Levels but Reduced Intratumoral Levels

Serum 25-HC increased with dose (220.94 ± 11.90, 240.16 ± 3.95, and 258.01 ± 7.35 μg/L in the vehicle, 1 mg/kg, and 10 mg/kg groups; one-way ANOVA: *F*(2,12) = 24.40, *p* < 0.0001; Tukey’s HSD: vehicle vs. 1 mg/kg, *p* = 0.0091; vehicle vs. 10 mg/kg, *p* < 0.0001; 1 vs. 10 mg/kg, *p* = 0.0145; Figure 1E). By contrast, intratumoral 25-HC (μg/mg protein) was lower in the 10 mg/kg group than in the vehicle group (12.92 ± 1.73 vs. 28.97 ± 4.43; *p* = 0.0001) and the 1 mg/kg group (31.10 ± 5.32; *p* < 0.0001; one-way ANOVA: *F*(2,12) = 29.11, *p* < 0.0001), whereas the 1 mg/kg group did not differ from the control (*p* = 0.6984; Figure 1F).

### 2.3. 25-HC Reduced Tumour-Cell Proliferation

**Figure 2:**
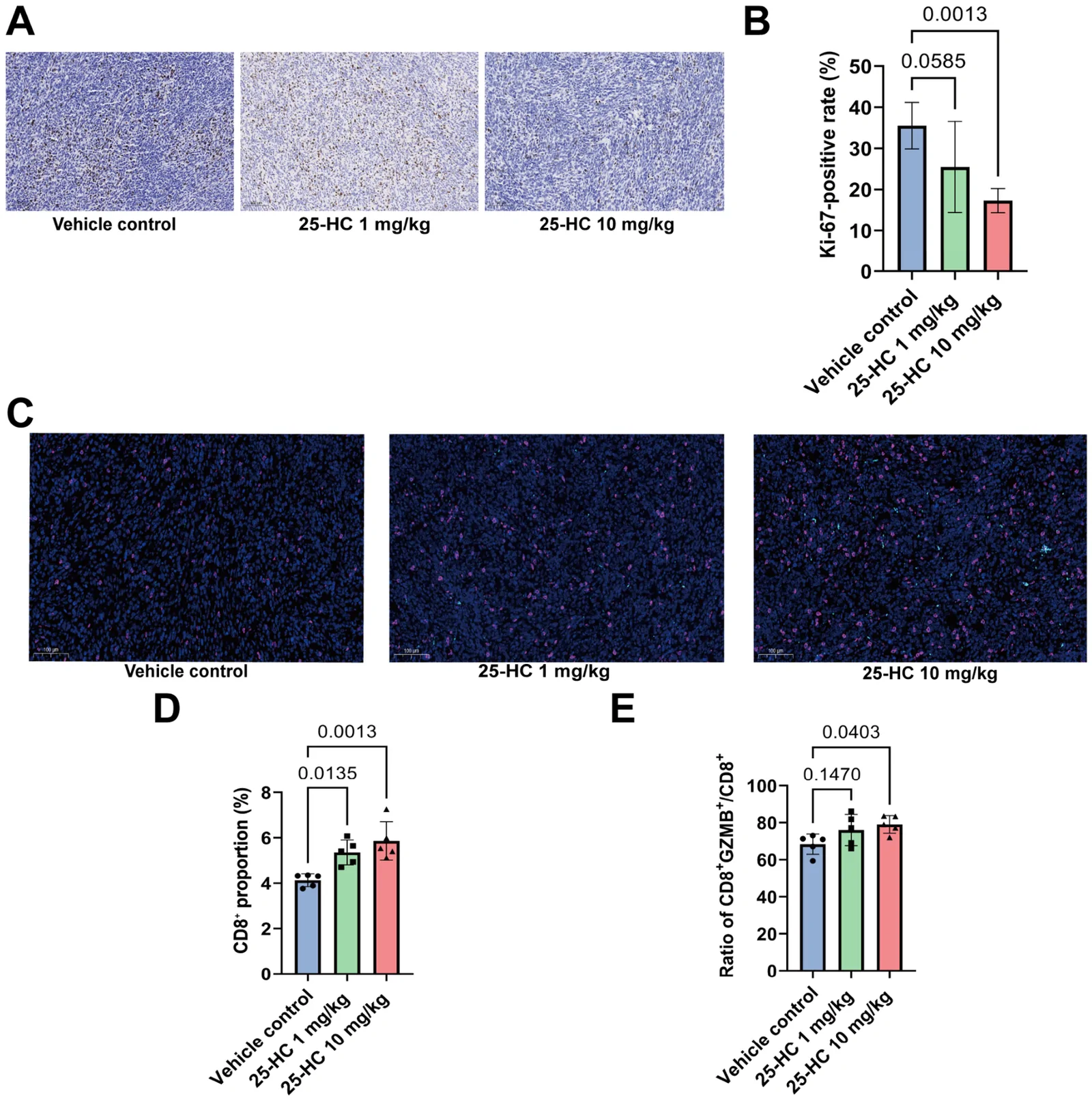
25-HC reduced tumour proliferation and enhanced CD8-positive T-cell responses. (**A**) Ki-67 immunostaining; (**B**) quantification of Ki-67-positive cells; (**C**) CD8/GZMB co-immunofluorescence; (**D**) CD8-positive proportion; (**E**) GZMB-positive proportion among CD8-positive cells. Data are mean ± SD (n = 5–6 per group); scale bars = 100 μm. One-way ANOVA followed by Dunnett’s post hoc test against the vehicle control: (**B**) Ki-67, adjusted *p* = 0.059 (1 mg/kg) and 0.0013 (10 mg/kg); (**D**) CD8, adjusted *p* = 0.0135 (1 mg/kg) and 0.0013 (10 mg/kg); (**E**) GZMB/CD8, adjusted *p* = 0.1470 (ns, 1 mg/kg) and 0.0403 (10 mg/kg); one-way ANOVA main effect for GZMB/CD8: *F*(2,12) = 3.65, *p* = 0.058.

Ki-67 positivity was quantified in three randomly selected ×200 fields per tumour section, with the mean per tumour used for analysis (n = 6 per group). Representative Ki-67 immunostaining is shown in Figure 2A. Ki-67-positive cells were 35.53 ± 5.67% (vehicle), 25.48 ± 11.08% (1 mg/kg) and 17.29 ± 2.94% (10 mg/kg) (one-way ANOVA: *F*(2,15) = 9.18, *p* = 0.0025). The reduction was significant at 10 mg/kg (Dunnett’s adjusted *p* = 0.0013 vs. vehicle) but did not reach significance at 1 mg/kg (adjusted *p* = 0.059; Figure 2B).

### 2.4. 25-HC Increased Cytotoxic CD8-Positive T-Cell Features

Representative CD8/GZMB co-immunofluorescence images are shown in Figure 2C. The proportion of CD8-positive cells was 4.13 ± 0.28% (vehicle), 5.36 ± 0.55% (1 mg/kg) and 5.86 ± 0.84% (10 mg/kg); GZMB-positive cells among CD8-positive cells increased from 68.38 ± 5.49% to 76.02 ± 8.44% and 79.05 ± 4.78% across the same groups. One-way ANOVA followed by Dunnett’s test identified significant increases in tumour CD8-positive-cell infiltration (*F*(2,12) = 10.95, *p* = 0.0020; adjusted *p* = 0.0135 and *p* = 0.0013 for 1 and 10 mg/kg vs. vehicle) and in the proportion of GZMB-positive cells among CD8-positive cells at 10 mg/kg (adjusted *p* = 0.0403; overall ANOVA *p* = 0.058, and adjusted *p* = 0.147 at 1 mg/kg; Figure 2D,E).

### 2.5. 25-HC Shifted Tumour Macrophages Away from an M2-Dominant State

**Figure 3:**
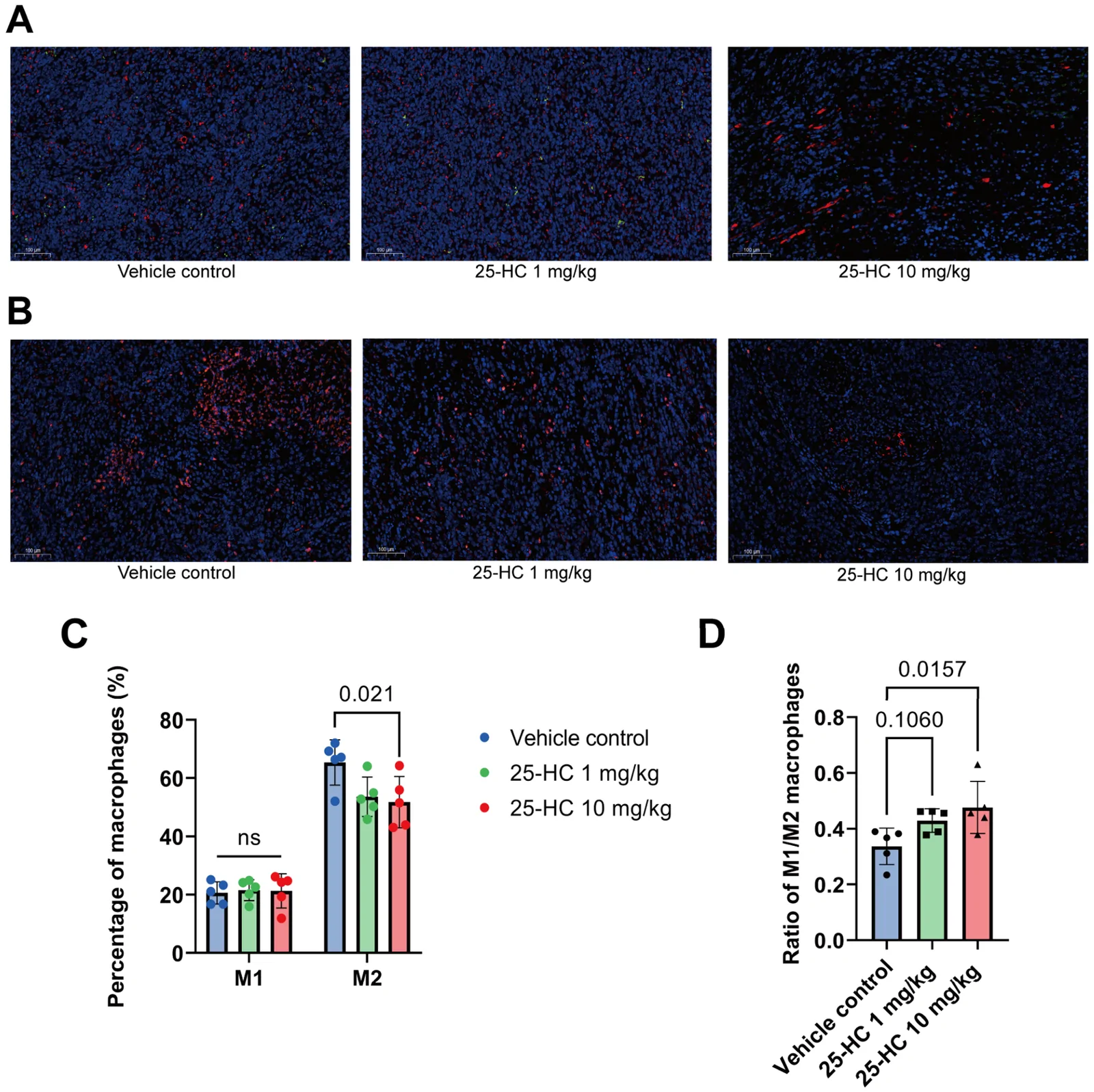
25-HC shifted tumour macrophages away from an M2-dominant state. (**A**) CD68/iNOS (M1-like) and (**B**) CD68/CD206 (M2-like) co-immunofluorescence; (**C**) percentages of iNOS-positive (M1-like) and CD206-positive (M2-like) cells among CD68-positive macrophages; (**D**) M1/M2 ratio. Five ×200 fields per tumour quantified (n = 5 per group). Scale bar = 100 μm. Bars show mean ± SD.

Representative co-immunofluorescence images are shown in Figure 3A,B. M1-like macrophages represented 20.62 ± 3.79%, 21.58 ± 3.61% and 22.52 ± 3.61% of macrophages in the vehicle, 1 mg/kg and 10 mg/kg groups, respectively (one-way ANOVA: *p* = 0.72; no significant between-group differences); M2-like macrophages represented 65.40 ± 7.76%, 53.62 ± 6.77% and 49.87 ± 9.85% (*F*(2,12) = 4.85, *p* = 0.029; Dunnett’s adjusted *p* = 0.021 for 10 mg/kg vs. vehicle, adjusted *p* = 0.076 at 1 mg/kg). The M1/M2 ratio increased from 0.34 ± 0.07 to 0.43 ± 0.04 and 0.48 ± 0.09 (*F*(2,12) = 5.11, *p* = 0.025; Dunnett’s adjusted *p* = 0.016 for 10 mg/kg vs. vehicle, adjusted *p* = 0.106 at 1 mg/kg; Figure 3C,D).

### 2.6. 25-HC Modulated Tumour AMPK/STAT6 Signalling

**Figure 4:**
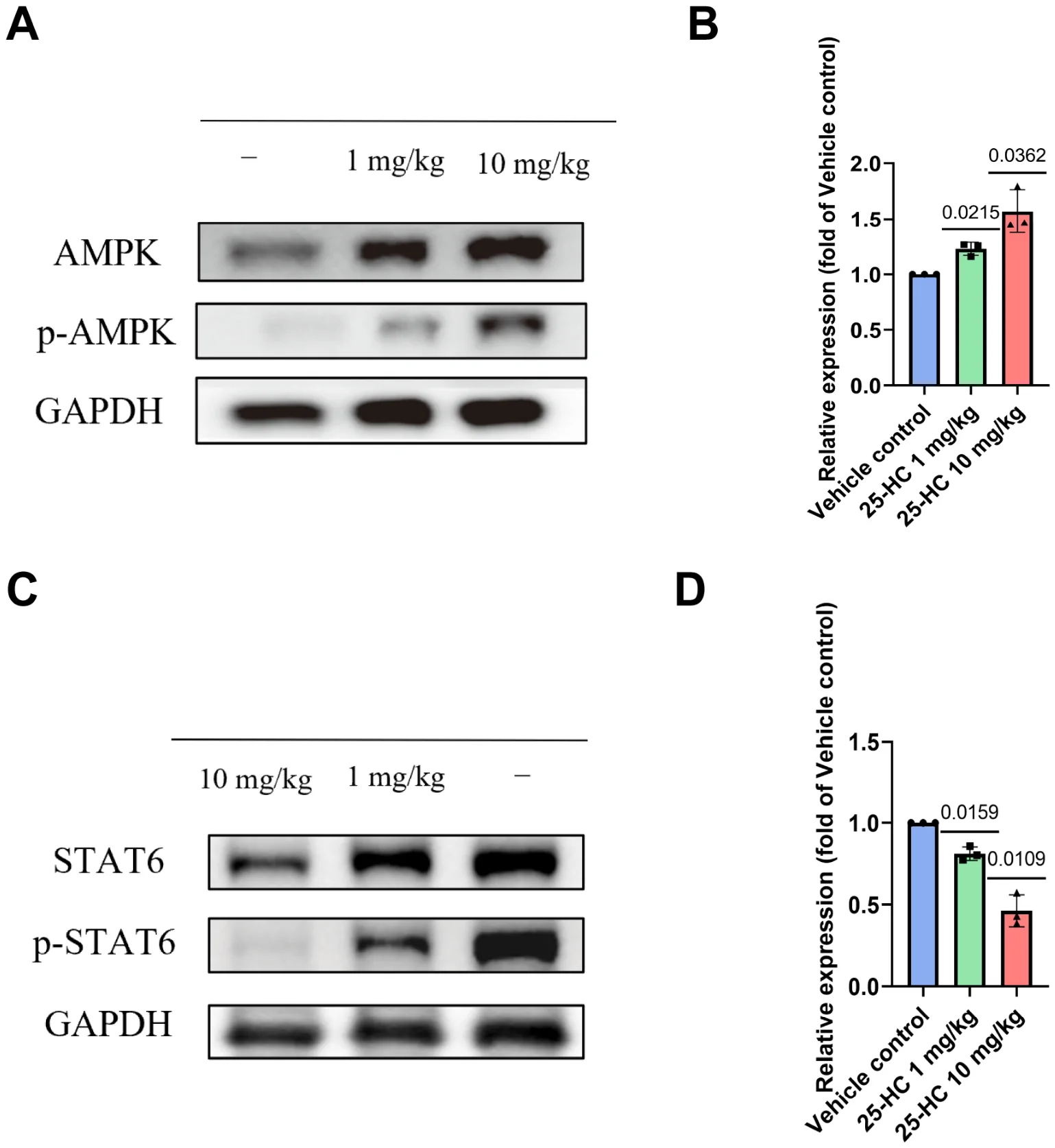
25-HC modulated tumour AMPK/STAT6 signalling. (**A**) Representative immunoblots of p-AMPK and total AMPK with GAPDH as loading control; (**B**) p-AMPK/AMPK ratio; (**C**) representative immunoblots of p-STAT6 and total STAT6 with GAPDH as loading control; (**D**) p-STAT6/STAT6 ratio. Values normalised to vehicle control (dashed line at 1.0); n = 3 independent biological replicates. (−) denotes vehicle control. One-sample *t*-test vs. 1.0.

Densitometric analysis (Figure 4) showed that p-AMPK/AMPK increased to 1.23- and 1.57-fold of the vehicle control at 1 and 10 mg/kg, respectively (*p* = 0.0215 and *p* = 0.0362, two-sided one-sample *t*-tests against 1.0; Figure 4A,B). Conversely, p-STAT6/STAT6 decreased to 0.81- and 0.46-fold of control (*p* = 0.0159 and *p* = 0.0109, respectively; Figure 4C,D).

### 2.7. The Antitumour Response to 25-HC Was Attenuated in BALB/c Nude Mice

**Figure 5:**
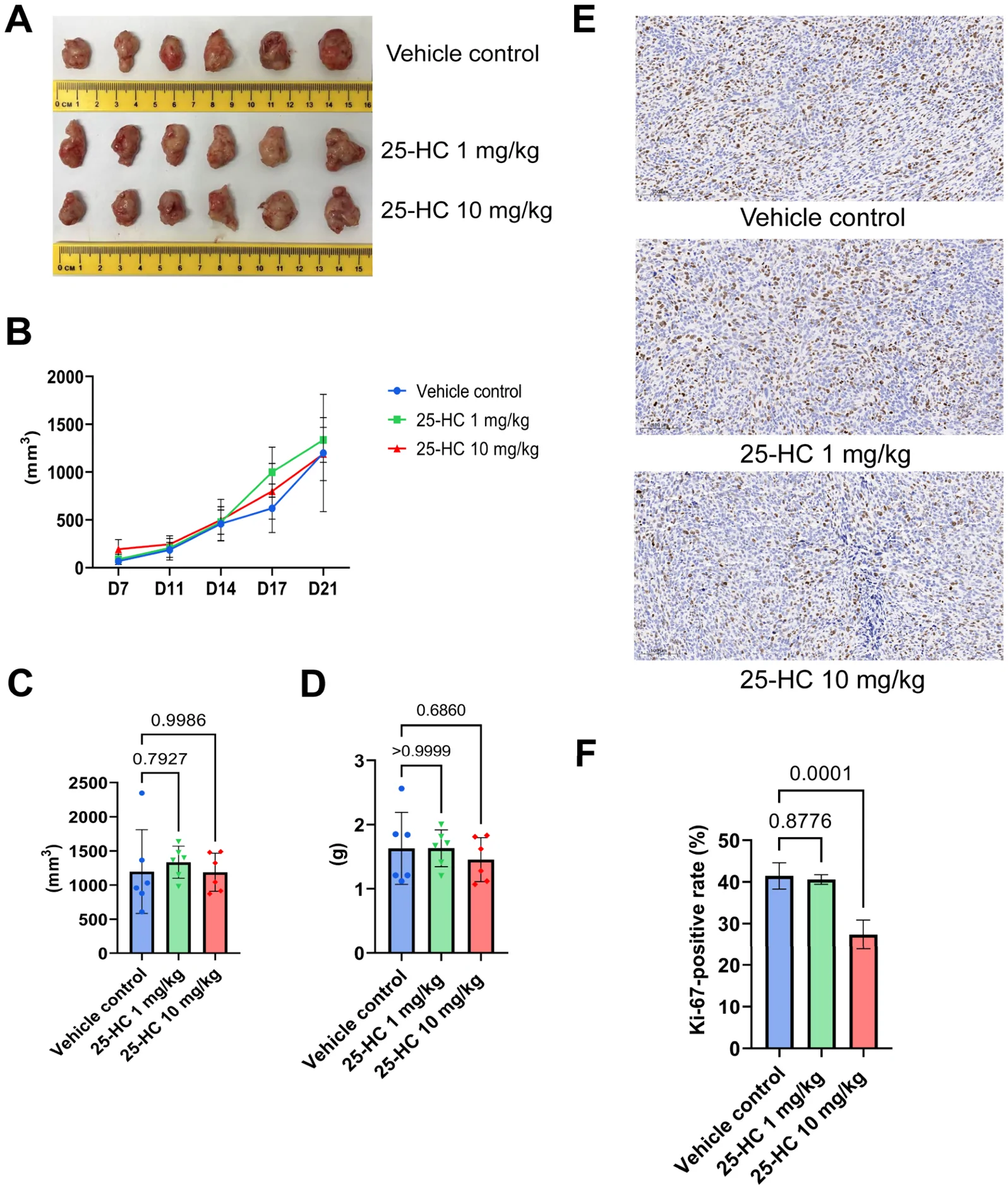
The antitumour response was attenuated in T-cell-deficient BALB/c nude mice. (**A**) Tumour images at endpoint; (**B**) tumour growth curves (days 7–21); (**C**) endpoint tumour volume (n = 6 per group); (**D**) tumour weight at endpoint (n = 6 per group); (**E**) Ki-67 immunostaining (×200; scale bar = 100 μm); (**F**) Ki-67 positivity (n = 4 per group). Bars show mean ± SD. One-way ANOVA followed by Dunnett’s test vs. vehicle: (**C**) adjusted *p* = 0.793 and 0.999 for 1 and 10 mg/kg, respectively; (**D**) adjusted *p* > 0.9999 and 0.686; (**F**) *F*(2,9) = 32.21, *p* < 0.0001; adjusted *p* = 0.8776 (1 mg/kg) and 0.0001 (10 mg/kg).

In the BALB/c nude-mouse arm (n = 6 per group), endpoint tumour volumes were 1198.9 ± 613.0 mm^3^ (vehicle), 1335.4 ± 234.2 mm^3^ (1 mg/kg), and 1188.7 ± 278.8 mm^3^ (10 mg/kg 25-HC). No dose-associated reduction was observed (one-way ANOVA: *F*(2,15) = 0.24, *p* = 0.79; Dunnett’s adjusted *p* = 0.793 and 0.999 for 1 and 10 mg/kg vs. vehicle, respectively; Figure 5A–C). Tumour weights were 1.63 ± 0.56, 1.64 ± 0.29, and 1.46 ± 0.34 g, respectively (one-way ANOVA: *p* = 0.70; Dunnett’s adjusted *p* > 0.9999 and 0.686 for 1 and 10 mg/kg vs. vehicle, respectively; Figure 5D). Ki-67 positivity was 41.4 ± 3.1%, 40.6 ± 1.2%, and 27.4 ± 3.5% (n = 4 per group; one-way ANOVA: *F*(2,9) = 32.21, *p* < 0.0001; Dunnett’s adjusted *p* = 0.8776 and 0.0001 for 1 and 10 mg/kg vs. vehicle, respectively; Figure 5E,F). To formally compare the treatment response between host strains, endpoint tumour volumes across both arms were re-analysed by a two-way factorial ANOVA with treatment (vehicle, 1 mg/kg, 10 mg/kg) and host (immunocompetent vs. nude) as factors. The treatment-by-host interaction did not reach statistical significance (*F*(2,30) = 3.02, *p* = 0.06), although the estimated direction was compatible with a possible host-context effect; the treatment main effect also did not reach statistical significance (*F*(2,30) = 2.98, *p* = 0.07), while the host main effect was not significant (*F*(1,30) = 0.36, *p* = 0.56).

## 3. Discussion

Oral 25-HC suppressed 4T1 tumour growth in immunocompetent BALB/c mice and remodelled the tumour immune microenvironment towards enhanced cytotoxic-effector features and reduced M2-like macrophage dominance, with reciprocal changes in tumour AMPK/STAT6 signalling. The attenuated response and the intermediate Ki-67 reduction in T-cell-deficient nude mice suggest that efficacy combines a direct antiproliferative component with an immune-context-associated component; efficacy differed only descriptively between host backgrounds, as the treatment-by-host interaction did not reach statistical significance (F(2,30) = 3.02, *p* = 0.06). These findings are discussed in detail below.

The intermediate Ki-67 reduction in the nude-mouse arm merits direct acknowledgement. Ki-67 positivity in nude mice fell by roughly one-third at the high dose, a reduction approaching the magnitude observed in immunocompetent animals, even though tumour volume and weight were not dose-dependently reduced in this arm. Of note, the nude-mouse arm showed markedly greater inter-animal variability than the immunocompetent arm, which limited the power of this arm to detect a modest effect. The lack of a statistically significant dose response should therefore be interpreted with caution rather than as definitive evidence of no effect. Importantly, because this antiproliferative change occurred in a T-cell-deficient host, the finding is compatible with a direct anti-proliferative effect of 25-HC on 4T1 cells, although it cannot be fully distinguished from effects mediated by residual host immune or stromal cells. The overall antitumour response may therefore reflect a combination of a direct antiproliferative effect on tumour cells and an immune-context-associated component. We acknowledge that this nuance sits in some tension with the study’s central argument, and that the present dataset cannot quantify the relative contribution of each component; distinguishing them would require further experiments, such as T-cell-depletion or adoptive-transfer studies.

The immune findings point to an immunomodulatory component of the observed antitumour activity. 25-HC-treated tumours contained more CD8-positive T cells and a greater fraction of GZMB-positive cells among CD8-positive T cells, while M2-like macrophage abundance decreased, and the M1/M2 ratio increased. Importantly, the macrophage data do not justify describing 25-HC as simply converting all tumour macrophages into M1 cells: the strongest evidence is a reduction in M2 dominance, whereas the absolute increase in M1-like macrophages was modest [11,12].

Notably, the direction of AMPK/STAT6 signalling observed here diverges from a recent report. Xiao et al. [4] showed that 25-HC activates lysosomal AMPKα and STAT6 in tumour-associated macrophages to promote an immunosuppressive, M2-like phenotype, whereas in the present tumour-tissue extracts p-AMPK increased while p-STAT6 decreased. Several non-mutually-exclusive mechanisms could explain why the two limbs dissociate in the present setting. First, because the measurements were obtained from whole tumour lysates, the two signals may originate from different cell populations: p-AMPK can increase in tumour cells or activated T cells as well as in macrophages, whereas STAT6 phosphorylation is a direct readout of interleukin (IL)-4/IL-13-driven M2 polarisation, and the net decline in p-STAT6 parallels the reduced CD206-positive M2-like macrophage abundance in the same tumours. Second, AMPK activation is strongly context-dependent and can either reinforce or counter-regulate inflammatory output in macrophages depending on dose, duration, subcellular compartment and concurrent STAT6 signalling [13,14]; STAT6–Trim24 crosstalk further fine-tunes M2 polarisation [15], and negative regulators such as SOCS1 may further restrain STAT6 signalling [16]. Third, the exposure context differs: Xiao et al. [4] examined lysosomal accumulation of 25-HC within tumour-associated macrophages, whereas in the present study 25-HC was administered orally, and intratumoral concentrations were comparatively low, so the signalling outcome may differ with dose and subcellular handling. The present observations may therefore reflect a net, cell-mixed signal rather than a reversal of the macrophage programme described by Xiao et al. [4], and cell-type-resolved approaches are required to define the underlying circuitry. Other work demonstrates that 25-HC supports CD8+ T-cell effector function and is actively consumed by activated cytotoxic T lymphocytes (CTLs) [7]; modulating cholesterol metabolism in CD8+ T cells potentiates their antitumour response [17]. In pancreatic cancer, loss of CH25H downregulates major histocompatibility complex class I (MHC-I) and reduces intratumoral CD8+ T-cell infiltration [18], and tumour-derived factors downregulate dendritic-cell (DC) CH25H to impair cross-presentation [19]. The present dataset is consistent with a net shift towards enhanced T-cell effector function and reduced M2 dominance, but does not resolve the cell-type-specific mechanism.

**Figure 6:**
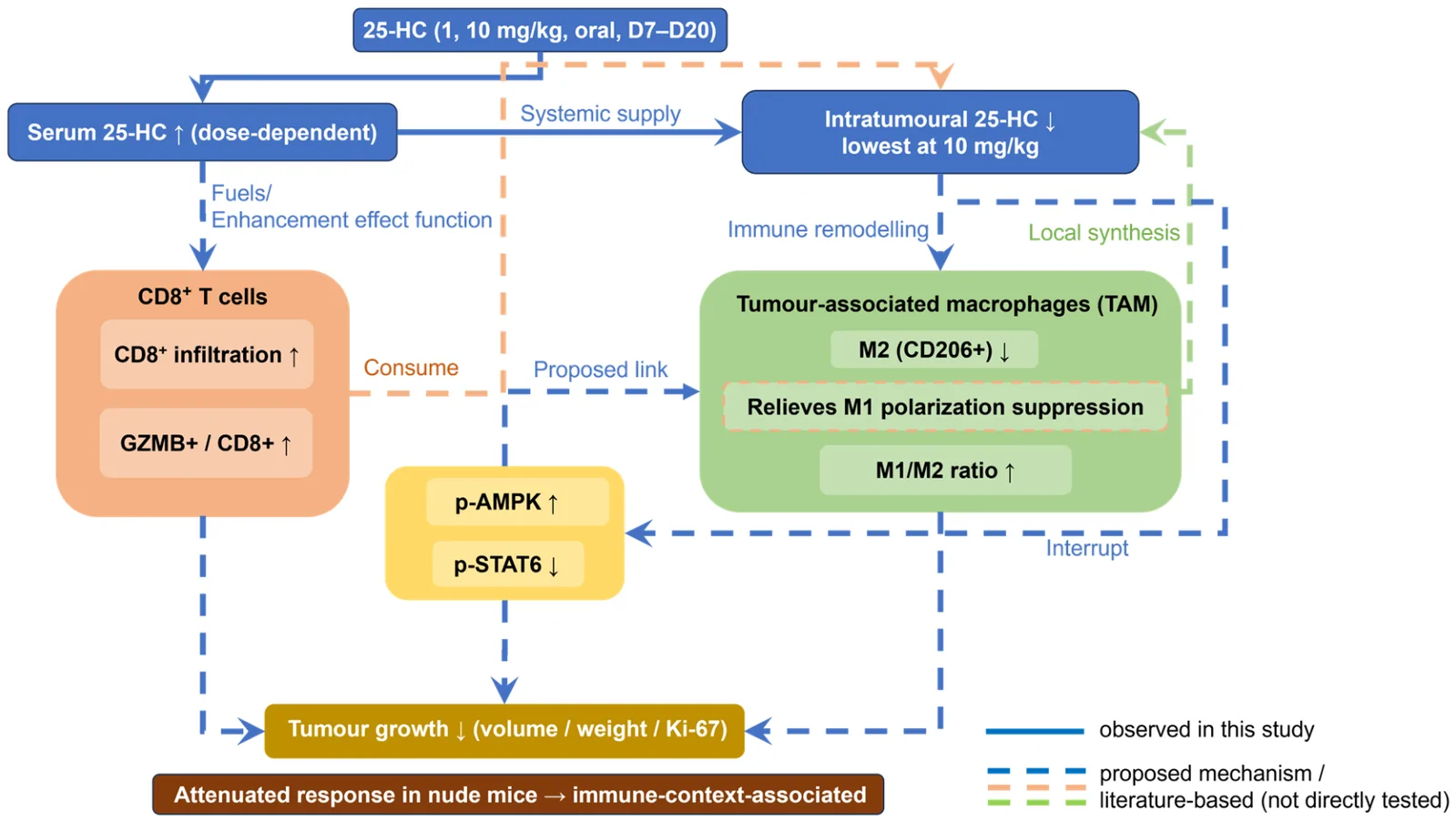
Proposed working model of oral 25-HC action in the 4T1 model. Oral 25-HC raised serum 25-HC (systemic supply), whereas intratumoral 25-HC was lowest at the highest dose. As serum and intratumoral 25-HC were measured at a single time point, the cause of this reciprocal serum–tumour pattern is unclear, and it is depicted here only as a working hypothesis of immune-cell-associated 25-HC handling within the tumour (a phenomenon of unclear cause), not as an established cellular mechanism. In the tumour microenvironment, CD8-positive T cells are hypothesised to consume 25-HC as a metabolic fuel (as proposed in the literature), tumour-associated macrophages may locally synthesise 25-HC, and the accompanying immune remodelling is characterised by reduced M2-like macrophage dominance, enhanced CD8/GZMB-positive cytotoxic features and increased p-AMPK with decreased p-STAT6 in bulk tumour lysates. Solid arrows indicate relationships observed in the present study; dashed arrows indicate proposed mechanisms based on the literature that were not directly tested and should not be interpreted as proven cellular events. Presented as a testable hypothesis; the responsible cell types and causal sequence remain to be confirmed.

A working model integrating these observations is presented in Figure 6 as a testable hypothesis rather than an established mechanism. The modest elevation of serum 25-HC may nonetheless be biologically relevant because systemic concentrations do not capture the pharmacologically active compartment: intratumoral 25-HC was lowest at the highest dose. However, because serum and intratumoral 25-HC were measured at a single time point, the cause of this reciprocal serum–tumour pattern cannot be determined; it should therefore be regarded as a descriptive observation of unclear cause rather than as evidence for immune-cell-associated handling of 25-HC. Whether immune cells take up or consume 25-HC within the tumour remains an untested working hypothesis. The responsible cell types and causal sequence remain to be confirmed, and definitive assessment of whether a systemic shift of this size drives the observed tumour inhibition would require pharmacokinetic–pharmacodynamic studies.

This study has several limitations. First, the dataset was small, with six animals per group for tumour-growth endpoints, five per group for immune measurements, three per group for western blotting, and four per group for nude-mouse Ki-67 assessment. In addition, although nude mice are T-cell-deficient, they retain innate immune components, including natural killer (NK) cells and macrophages, and therefore do not represent a pure T-cell-absence model; residual innate immunity may have contributed to the antiproliferative changes observed in this arm. Second, 25-HC was measured by competitive ELISA, a screening-level assay whose analytical specificity for 25-HC over structurally related oxysterols was not independently verified in the present study. Third, oral pharmacokinetic parameters (bioavailability, half-life, elimination) were not determined, so the exposure–response relationship between systemic and intratumoral 25-HC could not be rigorously assessed. Fourth, the proposed “25-HC immune-consumption cycle” was not directly tested; p-AMPK and p-STAT6 were measured in whole tumour lysates and cannot be assigned to a specific cell type. Fifth, direct effects of 25-HC on 4T1 cells were not assessed in vitro within the scope of the present study, so a direct antiproliferative component cannot be fully distinguished from effects mediated by residual host immunity. Finally, the study did not include human samples, target-engagement measurements, or a clinically validated dose range.

Overall, the findings support further evaluation of 25-HC as a preclinical immunometabolic candidate in TNBC. The observed tumour inhibition, immune remodelling, reciprocal serum–tumour 25-HC pattern, and AMPK/STAT6 changes generate a mechanistic hypothesis that warrants further investigation. Future studies with serial sampling, stable-isotope tracing, and independent liquid chromatography–tandem mass spectrometry (LC-MS/MS) confirmation will be required to test this model directly.

## 4. Materials and Methods

### 4.1. Syngeneic Tumour Model and Treatment

**Figure 7:**
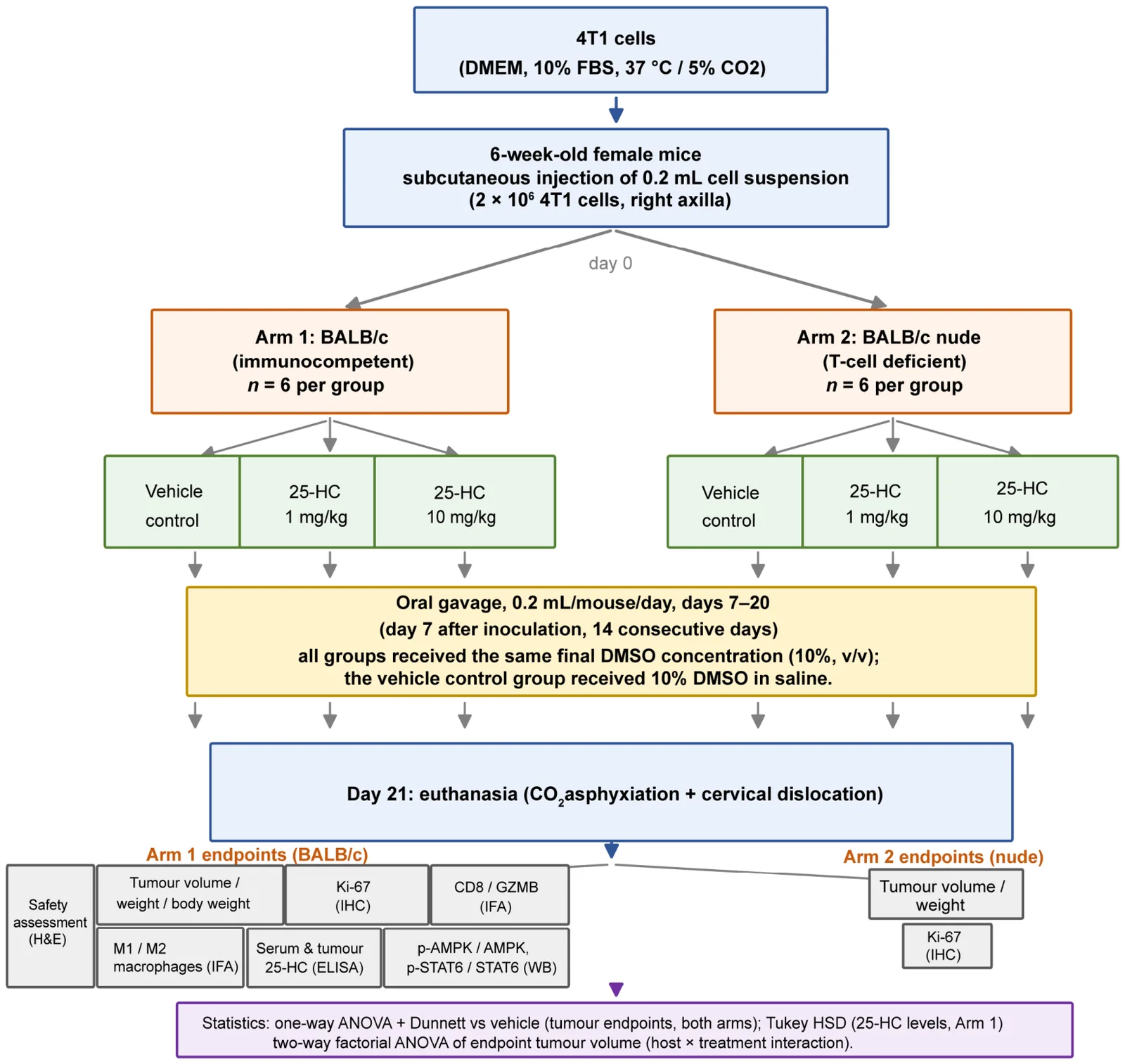
Experimental design. 4T1 cells were inoculated subcutaneously into the right axilla of female BALB/c mice. When tumours became palpable, mice were randomised to oral gavage of vehicle or 25-hydroxycholesterol (25-HC) at 1 or 10 mg/kg once daily for 14 days (n = 6 per group). Tumour volume was measured every 3–4 days. At endpoint, serum, tumour and selected organs were collected for enzyme-linked immunosorbent assay (ELISA), histology, immunohistochemistry, immunofluorescence and western blotting. A parallel arm using T-cell-deficient BALB/c nude mice followed the same treatment schedule.

The 4T1 mouse TNBC cell line(Cell Bank of Type Culture Collection, Chinese Academy of Sciences, Shanghai, China) was cultured in Dulbecco’s modified Eagle’s medium (DMEM, KeyGen Biotech, Nanjing, China) supplemented with 10% foetal bovine serum (Gibco, Grand Island, NY, USA) at 37 °C in a 5% CO_2_ humidified incubator; the medium was changed every 2–3 days, and cells were passaged at a ratio of 1:3 at approximately 80% confluence. Cells in the logarithmic growth phase (85–90% confluence) were washed with PBS (Biosharp, Hefei, China), detached with 0.25% Trypsin–EDTA (Gibco, Grand Island, NY, USA) for 1 min at 37 °C, neutralised with complete medium, collected by centrifugation (1000 rpm, 3 min), washed once with PBS (800 rpm, 4 min) and resuspended in PBS at 1 × 10^7^ cells/mL. Female BALB/c mice (6 weeks old), obtained from Nanjing Ziyuan Biological Co., Ltd., were inoculated subcutaneously with 0.2 mL of the cell suspension (2 × 10^6^ cells) into the right axilla to establish 4T1 tumours. Mice were assigned to a vehicle control, 1 mg/kg 25-HC (Felix Biotechnology Co., Ltd., Shanghai, China) or 10 mg/kg 25-HC group (n = 6 per group) by simple random allocation; no allocation concealment was applied. 25-HC was dissolved in dimethyl sulfoxide (DMSO, Sigma-Aldrich, St. Louis, MO, USA) at 10 mg/mL as a stock solution. Dosing formulations were prepared fresh daily, protected from light, and vortex-mixed immediately before administration, such that all groups received the same final DMSO concentration (10%, *v*/*v*) in a total gavage volume of 0.2 mL per mouse per day. Specifically, the 1 mg/kg dose contained 2 μL of stock solution supplemented with 18 μL pure DMSO and 180 μL sterile saline; the 10 mg/kg dose contained 20 μL of stock solution and 180 μL saline; and the vehicle control received 20 μL pure DMSO with 180 μL saline. Mice were dosed by oral gavage once daily. Treatment began on day 7 after model establishment and continued for 14 days (Figure 7); mice were euthanised on model day 21. Animals were euthanised by gradual CO_2_ inhalation in a sealed acrylic chamber, followed by cervical dislocation as a confirmatory method; no animals required early euthanasia during the study. Tumour measurements and endpoint assessments were performed without blinding to group allocation.

Tumour length and width were measured every 3–4 days. Tumour volume was calculated as V = ½ × a × b^2^, where a is the longest diameter, and b is the shortest diameter. Body weight was recorded during treatment. Tumours and selected organs were collected at endpoint for downstream analyses; representative haematoxylin and eosin (H&E)-stained sections of heart, liver and kidney are shown in Supplementary Figure S1.

### 4.2. T-Cell-Deficient (Nude) Mouse Arm

The same 4T1 model and treatment schedule were used in a separate arm of female BALB/c nude mice obtained from Nanjing Ziyuan Biological Co., Ltd. Six mice were included in each group (vehicle, 1 mg/kg 25-HC, and 10 mg/kg 25-HC). The inoculated cell number, administration route, allocation procedure, treatment schedule, tumour measurements and euthanasia procedure matched those used in the immunocompetent BALB/c arm (Table 1).

### 4.3. Histology, Immunohistochemistry and Immunofluorescence

Tumour tissues were fixed in 4% paraformaldehyde, embedded in paraffin and sectioned. Ki-67 (Abcam, Cambridge, UK; ab15580) immunostaining was used to estimate tumour proliferative activity [20]; three randomly selected high-power fields (×200) per tumour section were analysed, total cell numbers and Ki-67-positive cells were counted, and the mean positive rate per tumour was used for analysis, with the animal as the independent unit (n = 6 per group in the immunocompetent arm; n = 4 per group in the nude-mouse arm). CD8/granzyme B (GZMB, Abbkine, Wuhan, China; CY6795) co-immunofluorescence was used to assess cytotoxic T-cell infiltration and activation [21], and cluster of differentiation 68 (CD68, Boster Bio, Pleasanton, CA, USA; BA3638)/inducible nitric oxide synthase (iNOS, Proteintech, Rosemont, IL, USA; 18985-1-AP) and CD68/mannose receptor C-type 1 (CD206, Proteintech, Rosemont, IL, USA; 18704-1-AP) co-staining to estimate M1-like and M2-like macrophage populations, respectively; five randomly selected fields (×200; scale bar = 100 μm) per tumour section were quantified, and the proportions of GZMB-positive cells among CD8-positive cells and of iNOS-positive (M1-like) and CD206-positive (M2-like) cells among CD68-positive cells were calculated, with the animal as the independent unit (n = 5 per group). Quantification was performed using ImageJ 1.x (NIH, Bethesda, MD, USA); thresholds were predefined and applied uniformly across all sections.

### 4.4. Western Blotting

Total protein was extracted from 4T1 tumour tissue, separated by sodium dodecyl sulphate–polyacrylamide gel electrophoresis (SDS-PAGE), transferred to polyvinylidene difluoride (PVDF) membranes, and detected by chemiluminescence. Each sample was loaded in triplicate on the same gel (technical replicates); three independent biological replicates (a subset of three animals per group; n = 3) were analysed. Densitometric values were averaged across technical replicates within each blot before between-group comparison. Band intensities were quantified as integrated density using ImageJ. Phosphorylated-protein signals were normalised to the corresponding total protein. Total protein bands were normalised to glyceraldehyde 3-phosphate dehydrogenase (GAPDH; loading control) before phospho/total ratios were calculated. The primary antibodies were rabbit anti-phospho-AMPKα1/2 (Thr183/172; ImmunoWay, YP0575; 1:1000), rabbit anti-AMPKα1 (PT0166; ImmunoWay, YM8099; 1:3000), rabbit anti-STAT6 (PT0623R; ImmunoWay, YM8432; 1:5000), rabbit anti-phospho-STAT6 (Tyr641; A16N6; Selleckchem, F0555; 1:1000), and mouse anti-GAPDH (Proteintech, 60004-1-Ig; 1:30,000).

### 4.5. ELISA for 25-HC Quantification

Serum and tumour tissues were collected at euthanasia. 25-HC concentrations in serum and tumour homogenates were measured using a commercially available competitive enzyme-linked immunosorbent assay (ELISA) kit (Jiangsu Sumeike Biological Technology Co., Ltd., Nanjing, China; Meike brand, catalogue no. MK0348OA; lot no. 202501) according to the manufacturer’s instructions. The quality-control data for this kit (lot no. 202501) were as follows: limit of detection, 0.25 μg/L; standard-curve *R*^2^, 0.9985; intra- and inter-assay coefficients of variation, 6.3% and 8.5%; recovery, 90.7%; and specificity, 98.4%. Serum samples were assayed at a 1:5 dilution in triplicate. For tumour tissues, lipids were extracted using a modified Bligh–Dyer method [22] before ELISA: approximately 50 mg of fresh tissue was homogenised at 1:20 (*w*/*v*) in ice-cold chloroform:methanol (2:1, *v*/*v*) containing 0.01% butylated hydroxytoluene (BHT) and centrifuged at 4 °C and 2000 g for 15 min; the lower organic phase was collected, dried under a gentle stream of nitrogen, reconstituted in the assay diluent provided by the manufacturer, and assayed in triplicate. For bicinchoninic acid (BCA) protein quantification, parallel tumour tissue samples were lysed separately, and total protein was measured in the resulting lysates. Serum 25-HC is expressed as μg/L; tumour 25-HC is normalised to total protein and expressed as μg per mg of total protein.

### 4.6. Statistical Analysis

Statistical analyses were performed using GraphPad Prism 9.5 and IBM SPSS Statistics 26.0. Data are presented as mean ± standard deviation (SD) unless otherwise stated. The animal was the independent experimental unit for in vivo endpoints; field-level measurements were averaged within each animal before analysis. Comparisons among three groups within each host arm were analysed by one-way analysis of variance (ANOVA). Serum and intratumoral 25-HC concentrations were compared using Tukey’s HSD test; other prespecified comparisons with the vehicle group used Dunnett’s multiple-comparisons test. To formally evaluate whether the treatment effect differed by host background, endpoint tumour volumes across the immunocompetent and nude arms were re-analysed by a two-way factorial ANOVA with treatment (vehicle, 1 mg/kg, 10 mg/kg) and host (immunocompetent vs. nude) as factors, including the treatment-by-host interaction term. For western blotting, each value represented the mean of three technical replicates on one blot, with three independent biological replicates per group (n = 3). Phosphorylated-to-total protein ratios were expressed relative to the vehicle control and compared with a one-sample *t*-test. Statistical significance was set at *p* < 0.05. The numbers of animals analysed per endpoint were predefined at the design stage and applied uniformly across all groups within each endpoint: six per group for tumour-growth endpoints and Ki-67 immunostaining in the immunocompetent arm, five per group for dual-marker immunofluorescence, four per group for nude-mouse Ki-67 assessment, and three per group for western blotting; the animal was the independent experimental unit in all analyses.

## 5. Conclusions

Oral 25-HC suppressed 4T1 tumour growth in immunocompetent BALB/c mice and was associated with reduced tumour-cell proliferation, remodelling of the tumour immune microenvironment (reduced M2-like macrophage dominance and enhanced cytotoxic-effector features), and altered tumour AMPK/STAT6 signalling. The attenuated response in BALB/c nude mice, together with the intermediate Ki-67 reduction in this arm, suggests that the antitumour efficacy is compatible with both a direct antiproliferative component and an immune-context-associated component, although the treatment-by-host interaction did not reach statistical significance (*p* = 0.06), and immune-context association remains suggestive rather than established. The reciprocal serum–tumour 25-HC pattern, a descriptive observation from a single time point, generates a testable hypothesis of immune-cell-associated 25-HC handling, which warrants further investigation.

## Figures and Tables

**Table 1:** Animal groups, treatment schedules and sample sizes. 25-HC, 25-hydroxycholesterol; CO_2_, carbon dioxide.

Arm	Mouse Strain	Group	n	25-HC Dose and Route	Treatment Schedule	Euthanasia
Arm 1 (immunocompetent)	BALB/c	vehicle/25-HC 1 mg/kg/25-HC 10 mg/kg	6 per group	Oral gavage, 0.2 mL/day	Days 7–20 after inoculation (14 consecutive days)	Day 21: CO_2_ inhalation followed by cervical dislocation
Arm 2 (T-cell-deficient)	BALB/c nude	vehicle/25-HC 1 mg/kg/25-HC 10 mg/kg	6 per group	Oral gavage, 0.2 mL/day	Days 7–20 after inoculation (14 consecutive days)	Day 21: CO_2_ inhalation followed by cervical dislocation

Note: 2 × 10^6^ 4T1 cells (0.2 mL of a 1 × 10^7^ cells/mL suspension) injected into the right axilla on day 0. Allocation to groups was completely randomised without allocation concealment; investigators were not blinded to treatment. No animal required early euthanasia before the scheduled endpoint.

## Data Availability

The raw data supporting the conclusions of this article (tumour volume, tumour weight, body weight, Ki-67, CD8/GZMB and macrophage measurements) are available from the corresponding author on reasonable request and will be deposited in a public repository (figshare) before publication, together with full uncropped western-blot images and the image-analysis files.

## Supplementary Materials

The following supporting information can be downloaded at: https://www.mdpi.com/article/10.3390/ijms27219735/s1.

## Abbreviations

25-HC, 25-Hydroxycholesterol; AMPK, Adenosine monophosphate-activated protein kinase; ATF3, Activating transcription factor 3; CH25H, Cholesterol 25-hydroxylase; CTL, Cytotoxic T lymphocyte; DMSO, Dimethyl sulfoxide; ER, Oestrogen receptor; GZMB, Granzyme B; HER2, Human epidermal growth factor receptor 2; M1, M1-like macrophage; M2, M2-like macrophage; NK, Natural killer cell; PBS, Phosphate-buffered saline; p-AMPK, Phosphorylated AMPK; p-STAT6, Phosphorylated STAT6; STAT6, Signal transducer and activator of transcription 6; TNBC, Triple-negative breast cancer; ANOVA, Analysis of variance; BCA, Bicinchoninic acid; BHT, Butylated hydroxytoluene; CD8, Cluster of differentiation 8; CD68, Cluster of differentiation 68; CD206, Mannose receptor C-type 1; DC, Dendritic cell; DMEM, Dulbecco’s modified Eagle’s medium; ELISA, Enzyme-linked immunosorbent assay; GAPDH, Glyceraldehyde 3-phosphate dehydrogenase; H&,E Haematoxylin and eosin; IL, Interleukin; iNOS, Inducible nitric oxide synthase; LC-MS/MS, Liquid chromatography–tandem mass spectrometry; MHC-I, Major histocompatibility complex class I; PVDF, Polyvinylidene difluoride; SDS-PAGE, Sodium dodecyl sulfate–polyacrylamide gel electrophoresis; SD, Standard deviation; TAM, Tumour-associated macrophage.
