# Supplementary material for: Oral 25-Hydroxycholesterol Suppresses 4T1 Tumour Growth in an Immune-Context-Associated Manner
Source: Int J Mol Sci. 2026 Oct 30;27(21):9735. doi: 10.3390/ijms27219735 (PMC13762521; doi:10.3390/ijms27219735)
Supplement: Supplementary file 1 [file ijms-27-09735-s001.zip › ijms-4606525-supplementary.pdf]

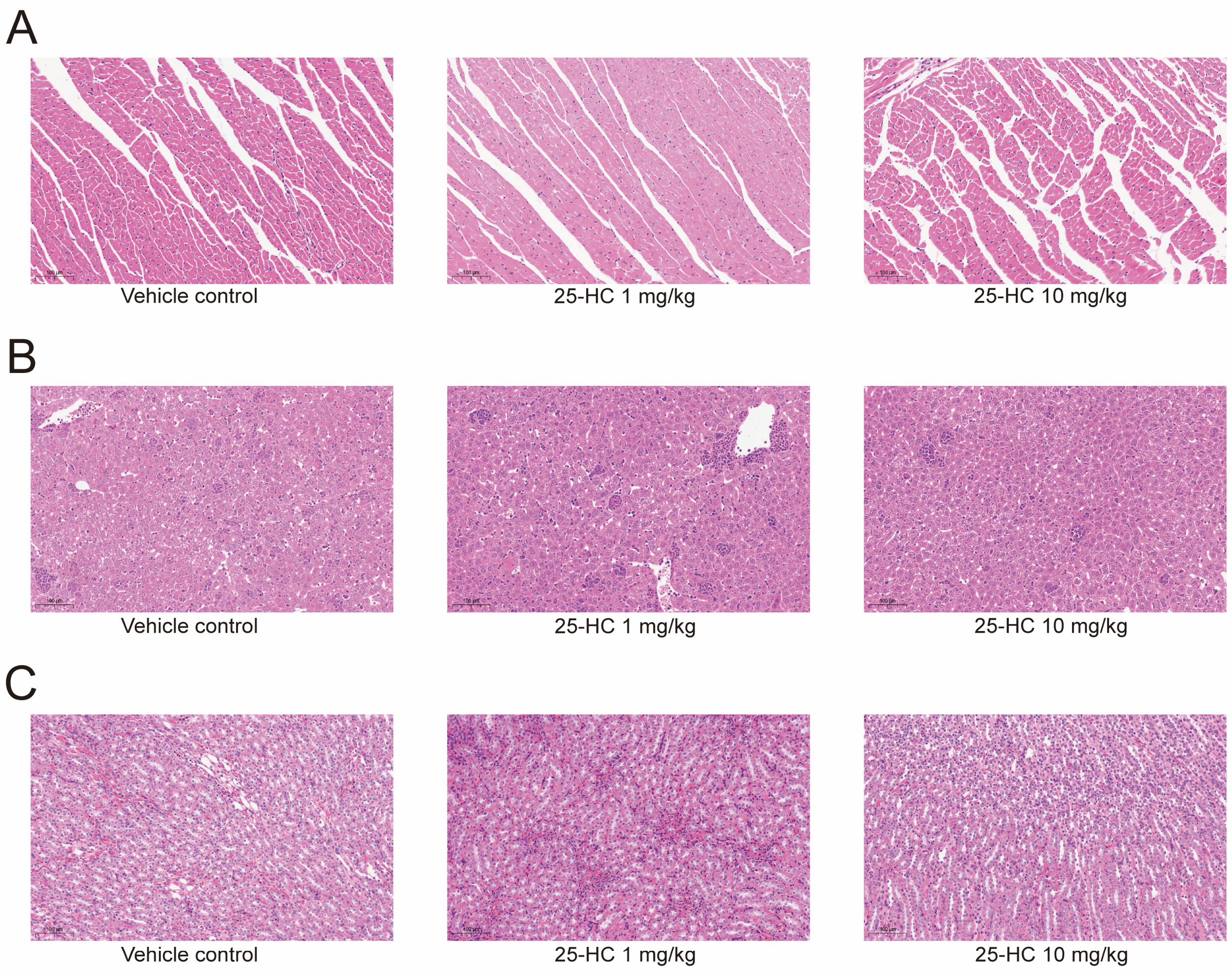

Figure S1. H&E staining of heart, liver and kidney in immunocompetent BALB/c mice 14 days after oral 25-HC treatment. Rows: (A) heart, (B) liver, (C) kidney; columns: vehicle, 1 mg/kg and 10 mg/kg (n = 6 per group; representative images, one per organ per group; scale bar = 100 μm). No treatment-related abnormalities were observed in the heart, liver or kidney.

Table Legends

Table S1. Animal groups, treatment schedules and sample sizes. 25-HC, 25-hydroxycholesterol; CO2, carbon dioxide.

**Arm**	**Mouse strain**	**Group**	**n**	**25-HC dose and route**	**Treatment schedule**	**Euthanasia**
Arm 1 (immunocompetent)	BALB/c	vehicle / 25-HC 1 mg/kg / 25-HC 10 mg/kg	6 per group	Oral gavage, 0.2 mL/day	Days 7–20 after inoculation (14 consecutive days)	Day 21: CO2 inhalation followed by cervical dislocation
Arm 2 (T-cell-deficient)	BALB/c nude	vehicle / 25-HC 1 mg/kg / 25-HC 10 mg/kg	6 per gr	Oral gavage, 0.2 mL/day	Days 7–20 after inoculation (14	Day 21: CO2 inhalation

	oup	consecutive days)	followed by cervical dislocation

Note: 2 × 10^6 4T1 cells (0.2 mL of a 1 × 10^7 cells/mL suspension) injected into the right axilla on day 0. Allocation to groups was completely randomised without allocation concealment; investigators were not blinded to treatment. No animal required early euthanasia before the scheduled endpoint.
